# An aquaporin mediates cell shape change required for cellular immunity in the beet armyworm, *Spodoptera exigua*

**DOI:** 10.1038/s41598-019-41541-2

**Published:** 2019-03-21

**Authors:** Shabbir Ahmed, Yonggyun Kim

**Affiliations:** Department of Plant Medicals, Andong National University, Andong, 36729 Korea

## Abstract

Cellular immunity in insects is accompanied by change in hemocyte shape. This study hypothesizes that cytoskeletal rearrangement is accompanied by transmembrane water transport to change cell volume, thus changing cell shape. A water-transporting pore (=aquaporin:AQP) has been identified in the beet armyworm, *Spodoptera exigua*. Its expression was detected in all developmental stages and tissues, although its transcription levels were different between biotic and abiotic conditions. Heterologous expression of *Se-AQP* in Sf9 cells showed that Se-AQP was localized on cell membrane. RNA interference (RNAi) using double-stranded RNA effectively suppressed its transcript levels. Under different ionic concentrations, hemocytes of RNAi-treated larvae did not change cell volume presumably due to malfunction in water transportation. Se-AQP might participate in glycerol transport because up-regulation of hemolymph glycerol titer after rapid cold-hardening was prevented by RNAi treatment against *Se-AQP* expression. The inhibitory effect of RNAi treatment on change of cell shape significantly impaired cellular immune responses such as phagocytosis and nodule formation upon bacterial challenge. RNAi treatment also significantly interfered with immature development of *S*. *exigua*. These results indicate that *Se-AQP* plays a crucial role in cell shape change that is required for cellular immunity and other physiological processes.

## Introduction

Aquaporins (AQPs) belong to major intrinsic proteins that form a diverse family consisting of more than 1,700 integral membrane proteins. They are responsible for transporting water and other neutral molecules through lipid bilayer membrane in almost all living organisms^1^. AQPs from both vertebrates and invertebrates have a similar structural organization with six transmembrane domains linked by five intra-helical loops. They are present as tetramer in the biological membrane. Each monomer of AQP contains two conserved Asn-Pro-Ala (NPA) signature motifs and an aromatic/arginine (Ar/R) constriction region for their selective permeability^2–4^.

There are 13 AQP genes denoted as AQP0-AQP12 in mammals. They are classified into two subfamilies^5,6^. The first subfamily is known as aquaporin that only permits water to pass through. The second subfamily is known as aquaglyceroporin that permits water and some other nonpolar small solutes such as urea and glycerol to pass through^7^. However, few AQPs have been discovered and characterized in insects. Phylogenetic analyses on insect AQPs have revealed the presence of six major subfamilies, including water/urea-transporting *Pyrocoelia rufa* integral proteins (PRIP), water-specific *Drosophila* intrinsic protein (DRIP), water and glycerol transporting aquaglyceroporin (Glp), glycerol-permeating entomoglyceroporin (Eglp), water-impermeable but cation-permeable Big Brain proteins (BIB), and unorthodox aquaporin (AQP12L)^8^.

Various roles of AQPs in mammalian systems have been reported. Besides their water transportation activity, they also play crucial roles in cell migration, cell proliferation, and adipocyte metabolism^9^. It has been reported that *AQP1*-deleted mice show growth retardation and reduced vascularity of implanted tumors^10^. Expression of *AQP4* in brain astrocytes can induce their migration toward a chemotactic stimulus and increase glial scarring^11,12^. However, expression of *AQP3* in skin and cornea can improve wound healing^13,14^ and colonic epithelial cell regeneration^15^. AQP7 and AQP9 have key metabolic regulatory functions in diabetes and obesity^16^.

In insects, AQPs play important roles in freeze tolerance, desiccation resistance, and heat tolerance^17–20^. An AQP identified from mosquito *Aedes aegypti* plays a crucial role in cell shape change by bidirectional water transport^21^. Change in cell shape is also required for cellular immune responses in insect immunity^22^. Especially, hemocytes exhibit spreading behavior to perform phagocytosis by increasing cell surface, in which cytoskeleton should be rearranged by F-actin growth and bundling^23^. Hemocyte-spreading behavior may also need cell volume change by bidirectional water transportation. Inhibition of water transport in hemocytes by treating ion channel inhibitor can impair hemocyte-spreading behavior^24^. The release of prophenoloxidase (PPO) for catalyzing melanization^25^ is also required for cell shape change to perform insect immunity. PPO is synthesized from a specific hemocyte, oenocytoid, and released into plasma by cell lysis in beet armyworm, *Spodoptera exigua*^26^. To trigger cell lysis, water should be transported into hemocytes presumably through AQP via an ion gradient established by sodium-potassium-chloride cotransporter activity^27^. Thus, the present study hypothesize that AQP activity is required for cell volume change of hemocytes to perform cellular immune responses.

In this study, an AQP gene (*Se-AQP*) was identified from *S*. *exigua*. Its expression patterns in different developmental stages and tissues including hemocytes were analyzed. After confirming its localization on cell membrane, physiological functions of *Se-AQP* associated with cellular immune responses were then assessed through RNA interference (RNAi).

## Results

### Molecular characterization and cellular location of Se-AQP

**Figure 1:**
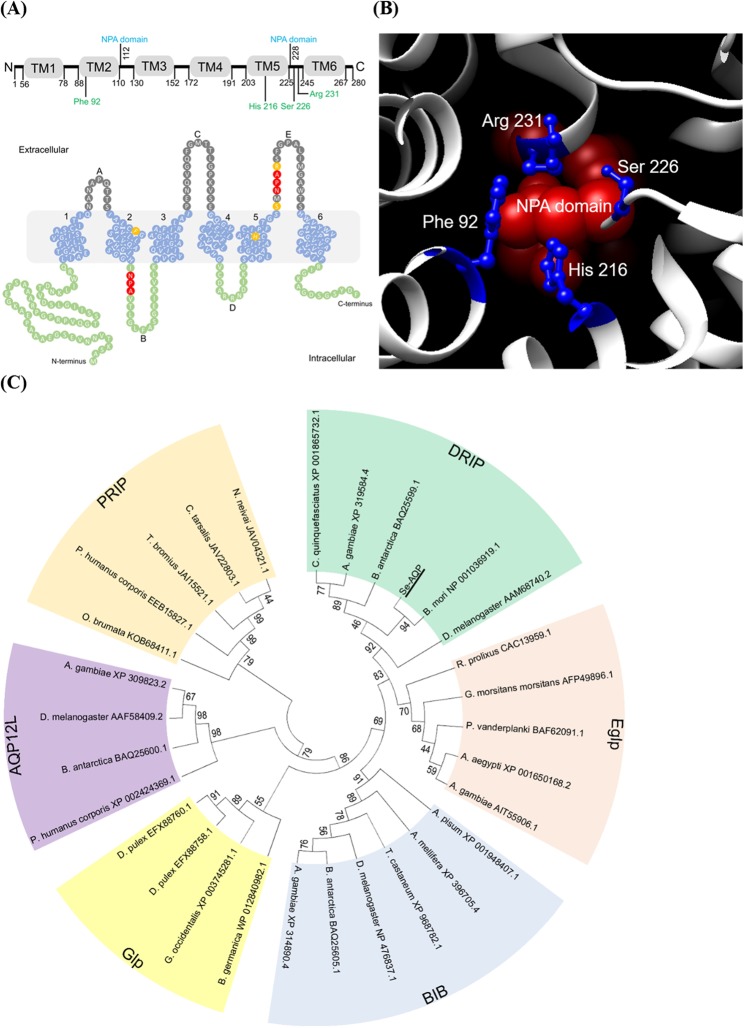
Molecular characterization of *S*. *exigua* aquaporin (Se-AQP). (**A**) Transmembrane domain analysis of Se-AQP. Domains of Se-AQP were predicted using TMHMM^69,70^. NPA domains are shown in red color while residues related to the Ar/R constriction region are shown in yellow color. (**B**) Organization of Ar/R constriction region. The structure depicted was from the extracellular side of the membrane. Classical NPA motifs are shown in red sphere. Ar/R selectivity residues regions (Phe-92, His-216, Ser-226, and Arg-231) are shown in blue balls and sticks. (**C**) Phylogenetic analysis of *S*. *exigua* aquaporin (Se-AQP, GenBank accession number: MH333284) with known insect AQPs. The analysis was performed using MEGA6. Bootstrapping values were obtained with 1,000 repetitions to support branching and clustering. Amino acid sequences of selected AQP genes were retrieved from GenBank. Accession numbers were added after species name.

*Se-AQP* was predicted from a TSA transcriptome (GenBank accession number: GAOQ01010693.1) by using *S*. *litura* aquaporin sequence (GenBank accession number: KC999953.1) as a query. Its ORF consists of 843 bp encoding 280 amino acids. *Se-AQP* domain analysis showed two tandem structural repeats, each consisting of three transmembrane helices (TM1-3 and TM4-6). It also had a short α-helix in loops B and E, each containing an NPA motif predicted to line one side of the pore (Fig. 1A) known as the “aquaporin fold”^28^. Residues responsible for the Ar/R constriction region (Phe-92, His-216, Ser-226, and Arg-231) were found in Se-AQP. They were predicted to have function of establishing water selectivity (Fig. 1B). For efficient water selectivity, Ar/R constriction region was present at close proximity to NPA domains. These conserved NPAs formed a canonical structure in the center of the pore^29^, allowing water molecule for passing through the midpoint of the channel^30^. Se-AQP appeared on the biological membrane as tetramer with each containing two NPA domains (Fig. S1A). Se-AQP shared 42.2% amino acid sequence similarities with *Homo sapiens* AQP (PDB accession number: 4CSK). Protein-protein interaction maps of Se-AQP with other proteins in *Drosophila melanogaster* were prepared due to no information on the interaction map of *S*. *exigua*. In this bioinformatics analysis, *Se-AQP* was predicted to interact with glycerol kinase (Fig. S1B). Phylogenetic analysis showed six clusters including PRIP, DRIP, Eglp, BIB, Glp, and AQP12L. *Se-AQP* was clustered with DRIP (Fig. 1C).

**Figure 2:**
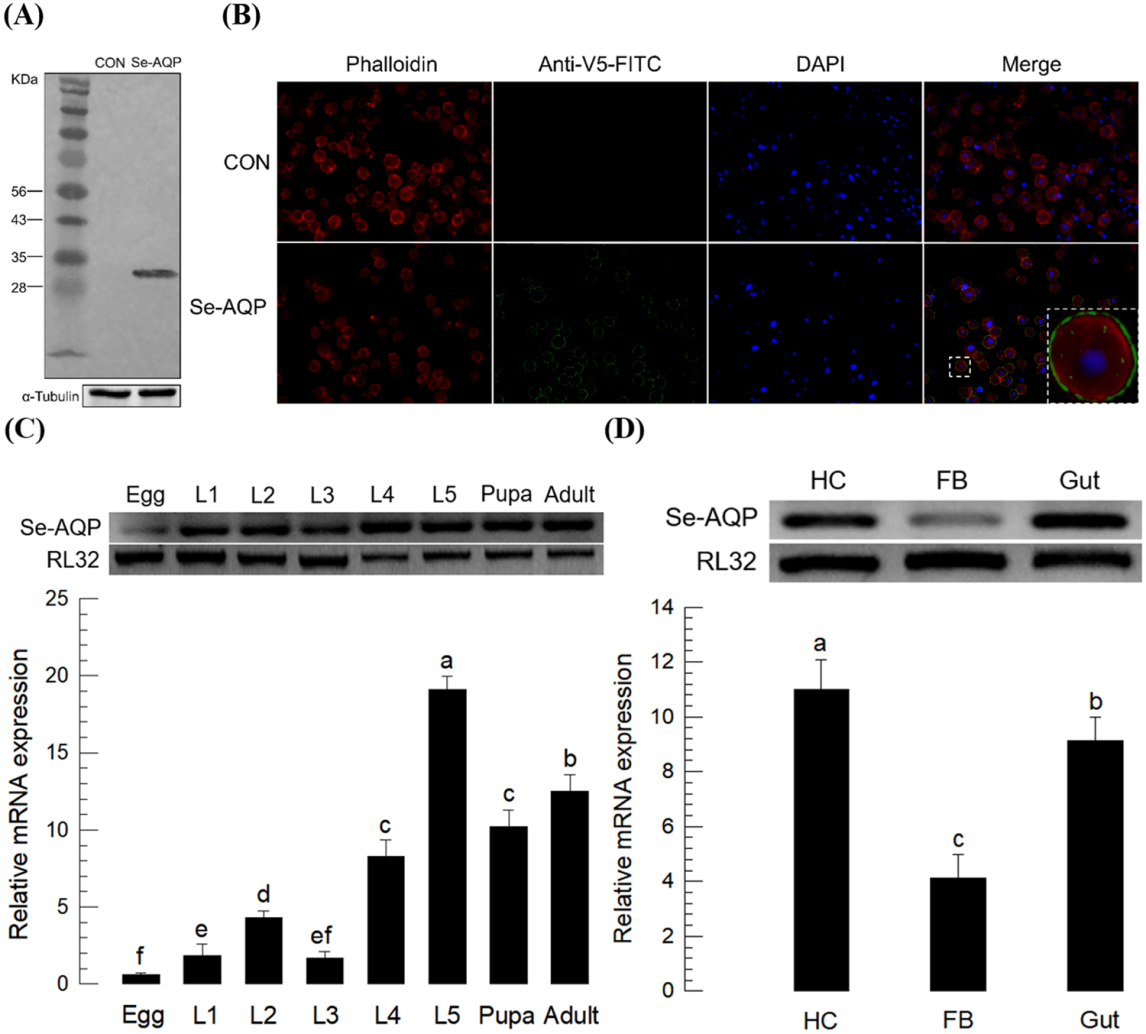
Expression profile of *Se-AQP*. (**A**) Western blot analysis. *Se-AQP* was transfected into Sf9 cells. Protein size of recombinant Se-AQP was ~32 kDa. It was captured by V5 antibody. (**B**) Immunofluorescence assay for the detection of transient expression of *Se-AQP* in Sf9 cells. F-actin was specifically detected with Alexa Fluor 555 phalloidin while nucleus was stained with DAPI. To check transient expression, anti-V5-FITC antibody was used. (**C**) Expression patterns of *Se-AQP* in different developmental stages, including egg, first to fifth instar larvae (‘L1–L5’), pupa, and adult. (**D**) Expression patterns in indicated tissues of L5 larvae, including hemocyte (‘HC’), fat body (‘FB’), and gut (‘Gut’). A ribosomal gene RL32 was used as reference gene. Each treatment was replicated three times with independent tissue preparations. Different letters indicate significant differences among means at Type I error = 0.05 (LSD test).

We transfected Sf9 cells with a eukaryotic expression vector containing *Se-AQP* to determine protein localization in expressed cells. Recombinant Se-AQP was heterologously expressed in Sf9 cells. The recombinant protein was confirmed by Western blot with expected size (~32 kDa) (Fig. 2A). Immunofluorescence assay showed that the recombinant Se-AQP protein was localized on the cell membrane of Sf9 cells (see a dotted big rectangle in Fig. 2B).

### *Se-AQP* expression and its down-regulation by RNAi

Expression of *Se-AQP* was analyzed under selected physiological conditions of *S*. *exigua*. *Se-AQP* was expressed in all developmental stages ranging from egg to adult, showing high expression levels during L5 larval and adult stages (Fig. 2C). Selected larval tissues were isolated and assessed for *Se-AQP* expression levels by RT-qPCR (Fig. 2D). *Se-AQP* exhibited the highest expression levels in hemocytes.

*Se-AQP* expression level was increased according to larval development, suggesting that its expression levels might be correlated with body size. To test this hypothesis, different body sized individuals in L4, L5, or pupal stage were assessed for expression levels of *Se-AQP*. Correlations between body weight and *Se-AQP* expression level were highly significant in L4 larvae (*r* = 0.88; *P* < 0.05), L5 larvae (*r* = 0.90; *P* < 0.05), and pupae (*r* = 0.70; *P* < 0.05) (Fig. S1A).

Body water content should be regulated according to physical climate conditions in insects. This raised a hypothesis that environmental factors such as temperature and humidity might influence the expression of *Se-AQP*. Results showed that expression levels of *Se-AQP* in larvae or pupae exposed to different temperatures for 6 h significantly fluctuated. They were different among developmental stages (*F* = 8.49; df = 2, 42; *P* < 0.001) (Fig. S1B). Expression levels of *Se-AQP* were significantly increased with increasing ambient temperature (*F* = 37.97; df = 1, 28; *P* < 0.001 for L4; *F* = 8.02; df = 1, 28; *P* < 0.01 for L5; *F* = 29.40; df = 1, 28; *P* < 0.001 for pupa). Humidity also influenced expression levels of *Se-AQP* in *S*. *exigua*. Their expression levels varied among developmental stages (*F* = 26.06; df = 2, 42; *P* < 0.001) (Fig. S2C). In both larval and pupal stages, *Se-AQP* expression levels were decreased with increasing relative humidity (*F* = 29.76; df = 1, 28; *P* < 0.001 for L4; *F* = 18.77; df = 1, 28; *P* < 0.01 for L5; *F* = 31.03; df = 1, 28; *P* < 0.001 for pupa).

RNAi was performed by injecting dsRNA specific to *Se-AQP* (Fig. S3). Test developmental stage was L5 larvae which took 5 days before pupation. The last 2 days of L5 larvae are considered a wandering phase for pupation while the first 3 days are considered its growing phase^31^. When 1 μg of dsRNA was injected to each of one day old L5 larvae, *Se-AQP* transcript levels were undetectable at 24 h PI in all three test tissues (Fig. S3A). Decreased levels of *Se-AQP* expression maintained at least for 3 days. To quantify RNAi efficacy, qPCR analyses were performed (Fig. S3B). dsRNA treatments resulted in significant reductions in *Se-AQP* expression in these tissues at 24 and 48 h PI. At 24 h PI, *Se-AQP* expression levels in hemocyte, fat body, and gut were decreased ~65.5%, ~84.1%, and ~68.8%, respectively. At 48 h PI, *Se-AQP* expression levels in hemocyte, fat body, and gut were decreased ~2.1, ~4.4, and ~14.4 fold, respectively, compared to controls. For subsequent functional analyses, dsRNA-treated larvae at 24 h PI were used.

### Roles of Se-AQP in transporting water and glycerol

**Figure 3:**
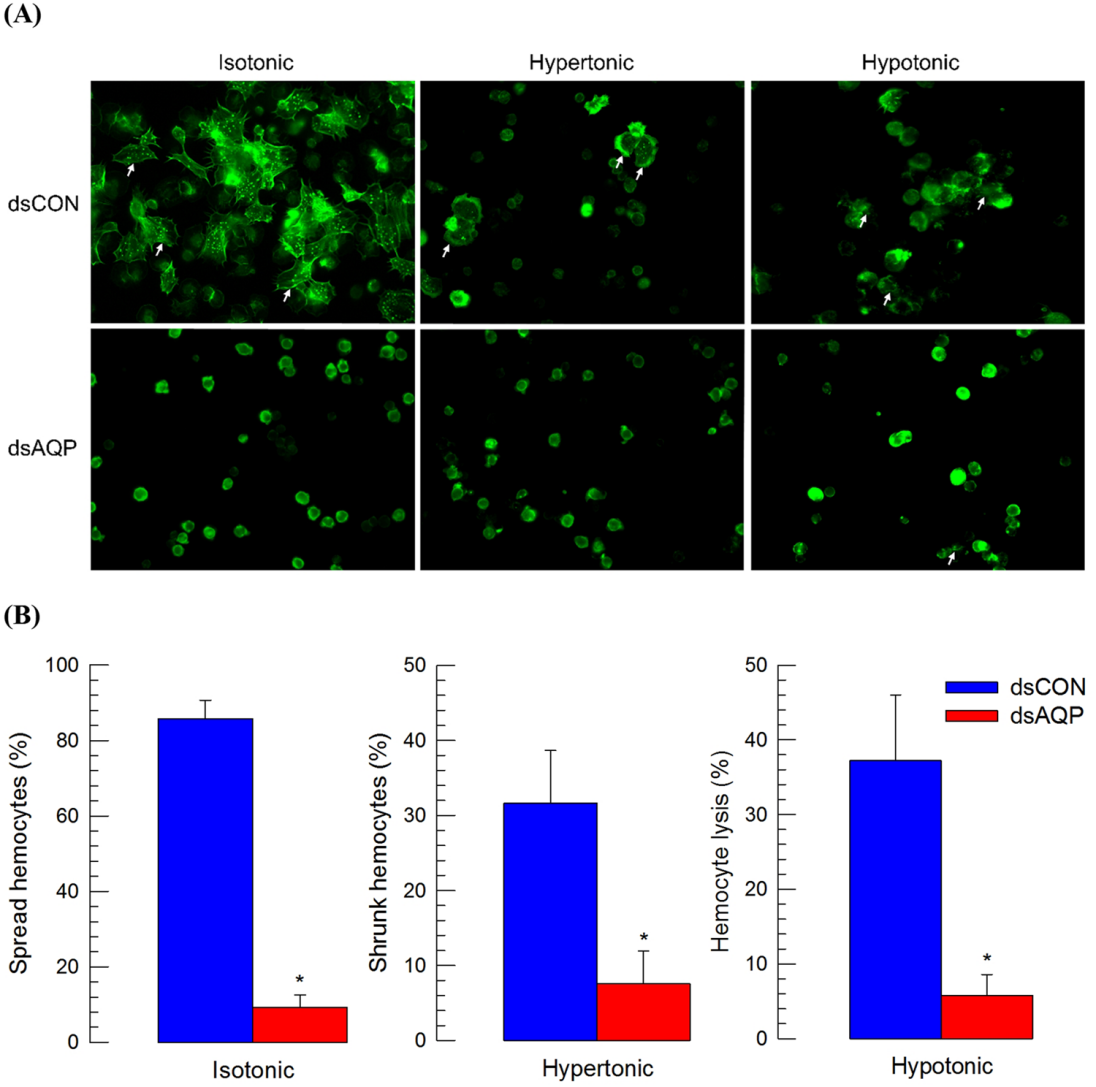
Role of Se-AQP in water transport. (**A**) Function of *Se-AQP* in regulating hemocyte shape. Hemocytes treated with gene specific dsRNA (‘dsAQP’) were exposed to isotonic, hypertonic, or hypotonic solution for 10 min. Hemocytes were observed under a fluorescence microscope at 400× magnification. Spread, shrunk, and lysed cells were indicated with white arrows. Hemocytic F-actin filaments were specifically recognized by FITC-tagged phalloidin (green). (**B**) Quantitative representation of spread, shrunk, and lysed hemocytes after exposure to isotonic, hypertonic, and hypotonic solutions, respectively. A GFP gene was used as a control dsRNA (‘dsCON’). Each treatment was independently replicated three times. Asterisk mark (*) on bars indicates significant differences among means at Type I error = 0.05 (LSD test).

To determine the role of Se-AQP in water transport, two groups of hemocytes were used to compare their cell shapes under different osmotic environments (Fig. 3). One group of hemocytes was prepared from larvae treated with dsAQP at 24 PI as shown above. The other group was control hemocytes prepared from larvae treated with control dsRNA. In controls, hemocytes showed spreading, shrunk, and lysed behaviors under isotonic, hypertonic, and hypotonic environments, respectively (Fig. 3A). Under isotonic environment, most hemocytes were well spread. Their cell-spreading behavior was determined by extension of F-actin out of original cell boundary. Under hypertonic environment, hemocytes were distorted and shrunk probably due to loss of water from cellular content. Under hypotonic treatment, most hemocytes were lysed probably due to water intake. However, hemocytes collected from RNAi-treated larvae significantly (*P* < 0.05) prevented these cell shape changes (Fig. 3B). Hemocytes under dsAQP treatment were ~10.6 fold less spread under isotonic condition, ~5.5 fold less shrunk under hypertonic condition, and ~8.6 fold less lysed under hypotonic condition compared to control hemocytes.

**Figure 4:**
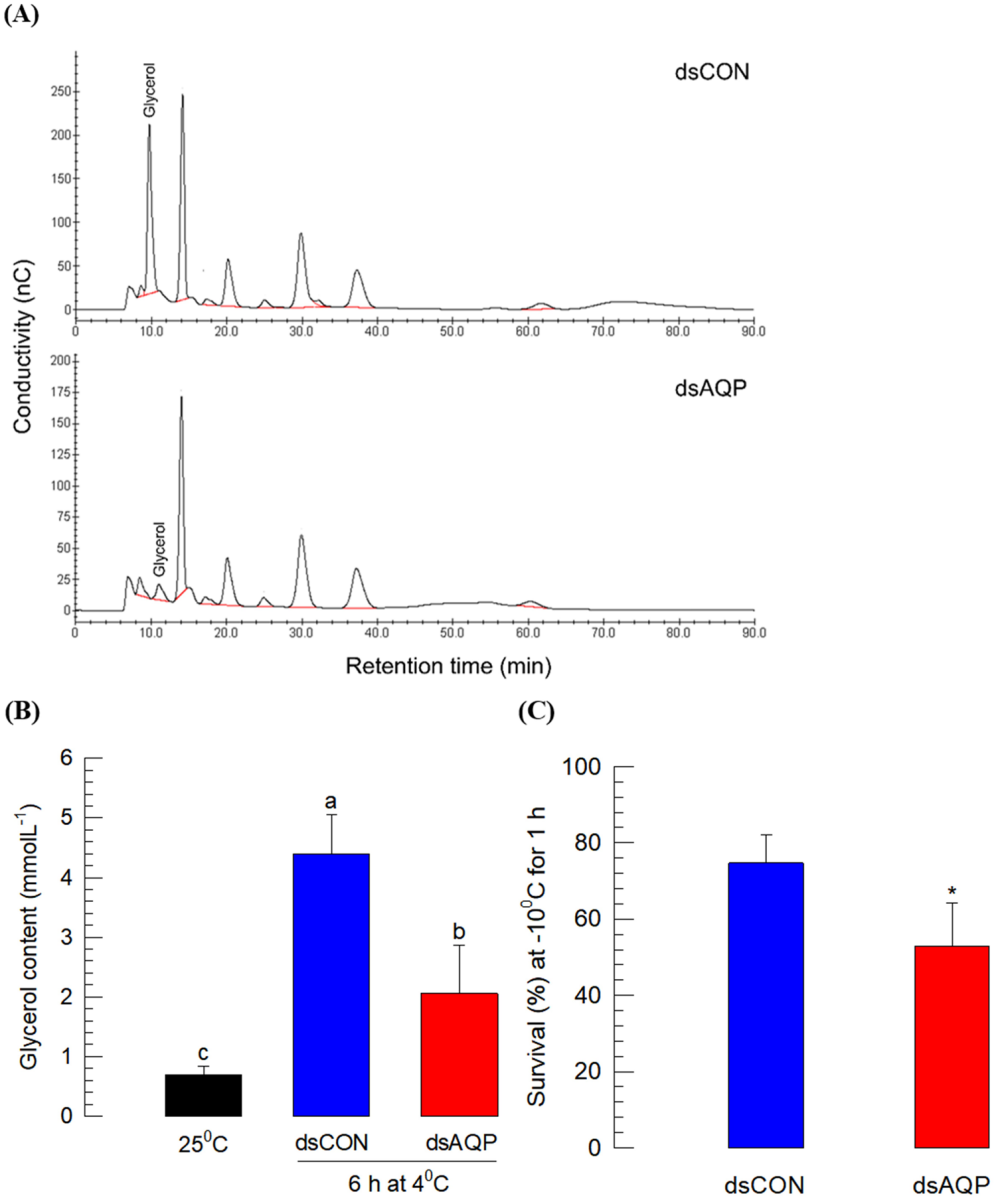
Role of Se-AQP in glycerol transport. (**A**) Chromatograms showing reduced glycerol titer in hemolymph of RNAi treated fifth instar larvae in response to exposure to 4 °C for 6 h. (**B**) Changes in glycerol content in fifth instar larval hemolymph in response to exposure to 4 °C for 6 h. The eluent was 400 mM NaOH at a flow rate of 0.4 mL/min. (**C**) Suppression of cold tolerance after RNAi treatment of *Se-AQP*. Each treatment was independently replicated three times. Each replicate used 10 larvae. Different letters and asterisk mark (*) indicate significant differences among means at Type I error = 0.05 (LSD test).

*S*. *exigua* is known to be freeze-susceptible. It needs rapid cold hardening (RCH) capacity for overwintering to supercool body water using cryoprotectant like glycerol^32^. Bioinformatics analysis indicated that *Se-AQP* was associated with glycerol kinase (Fig. S1B), suggesting that it might be involved in transport of glycerol during RCH. To test this hypothesis, RNAi was performed to knockdown expression of *Se-AQP* followed by RCH treatment (4 °C for 6 h) (Fig. 4). HPLC analysis showed that RCH treatment resulted in accumulation of glycerol in the hemolymph of control larvae. dsAQP treatment significantly decreased glycerol peak in the chromatogram (Fig. 4A). Results showed significant reduction (~2.6 fold) of glycerol content in the hemolymph of dsAQP-treated larvae after exposure to RCH treatment (Fig. 4B). This led us to determine whether Se-AQP might have a physiological function in cold tolerance. After RCH treatment, survivorship of RNAi-treated larvae was significantly reduced by ~1.5 folds compared to that of control larvae (Fig. 4C).

### Immune mediation by Se-AQP through cell shape change

**Figure 5:**
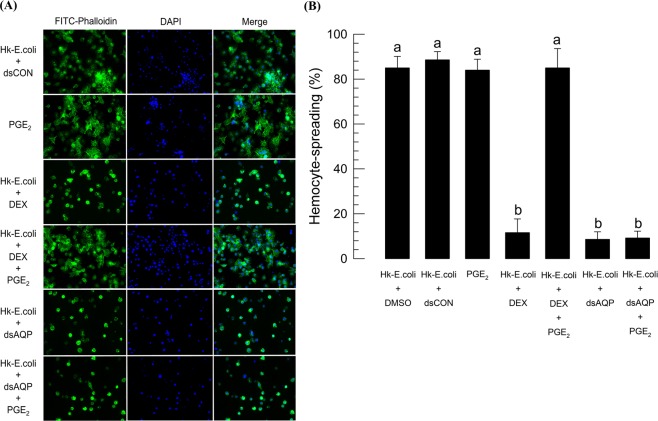
Role of Se-AQP in changing cell shape of hemocytes. For bacterial challenge, heat-killed (HK) *E*. *coli* (~3.2 × 10^4^ cells/larva) in 1 µL were injected into larvae at 24 h after dsRNA treatment. (**A**) Effect of dsAQP on F-actin growth in response to bacterial challenge. At 2 h PI, hemocytes were observed under a fluorescence microscope at 400× magnification. Hemocytic F-actin filaments were specifically recognized by FITC-tagged phalloidin (green) while the nucleus was stained with DAPI (blue). (**B**) Quantitative representation of hemocyte spreading assay. Each treatment was independently replicated three times. Different letters indicate significant differences among means at Type I error = 0.05 (LSD test).

Hemocytes were spread along with F-actin growth upon immune challenge using heat-killed *E*. *coli* (Fig. 5A). PGE_2_ injection was also effective in inducing the hemocyte-spreading behavior as much as bacterial challenge (Fig. 5B). However, treatment with dexamethasone (DEX, a PLA_2_ inhibitor) significantly (*P* < 0.05) inhibited the hemocyte-spreading behavior upon immune challenge. Addition of arachidonic acid (AA, a catalytic product of PLA_2_) significantly (*P* < 0.05) rescued the inhibitory activity of DEX against hemocyte-spreading behavior. Hemocytes collected from dsAQP-treated larvae also significantly (*P* < 0.05) lost their spreading behavior upon immune challenge and did not even respond to the addition of PGE_2_.

**Figure 6:**
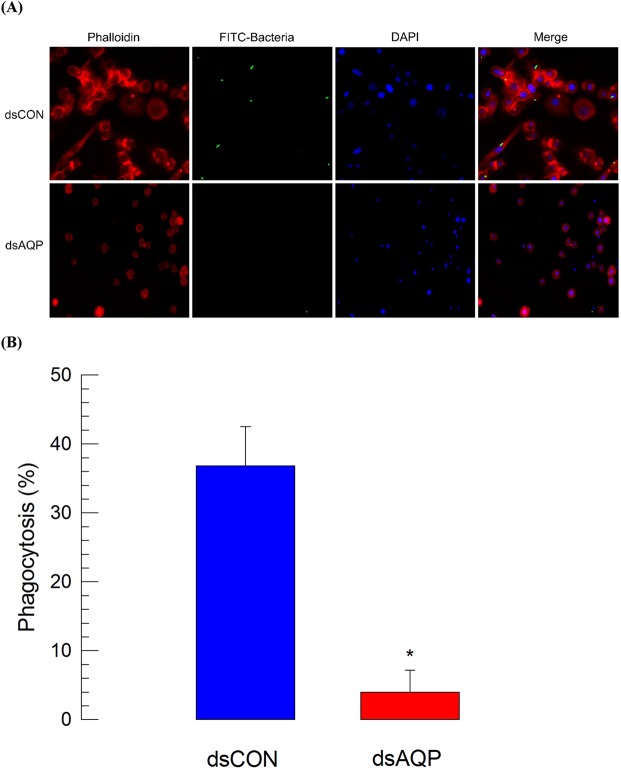
Influence of RNAi treatment of *Se-AQP* on hemocyte phagocytosis. (**A**) Effect of dsAQP on FITC-labeled *E*. *coli*. One microliter of heat-killed (HK) *E*. *coli* (~3.5 × 10^4^ cells/larva) were injected into larvae at 24 h after dsRNA treatment. A GFP gene was used as a control dsRNA (‘dsCON’). (**B**) Quantitative representation of phagocytosis between dsCON and dsAQP treated hemocytes. Each treatment was independently replicated three times. Asterisk mark (*) on bars indicates significant differences among means at Type I error = 0.05 (LSD test).

Cell shape change induced by Se-AQP activity might be required for cellular immunity of hemocytes in *S*. *exigua*. To test this hypothesis, hemocyte phagocytosis exhibiting cytoplasmic extension to form phagosome was assessed after injecting FITC-labeled *E*. *coli* to L5 larvae (Fig. 6). As expected, control hemocytes were well spread (Fig. 6A). Some (>35%) of hemocytes had FITC-labeled *E*. *coli* in their cytoplasm. However, phagocytosis was significantly (*P* < 0.05) lost in hemocytes collected from larvae treated with dsAQP, in which less than 4% hemocytes could perform cellular immunity (Fig. 6B).

**Figure 7:**
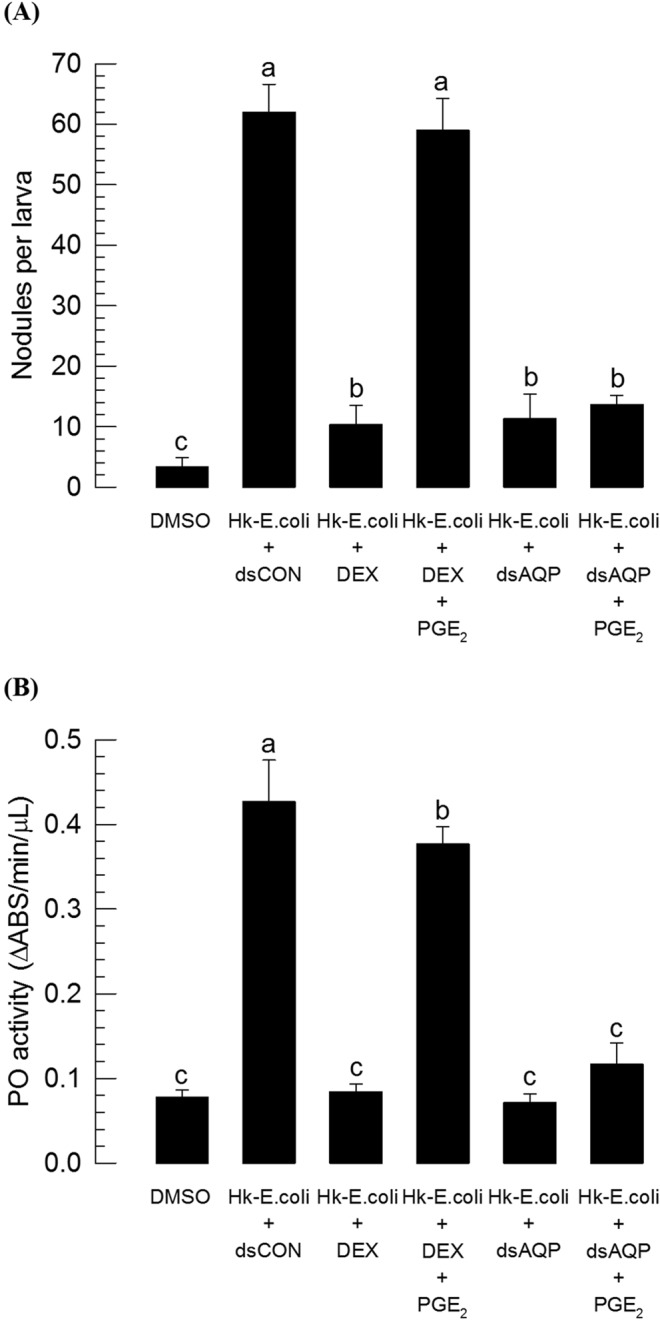
Influence of RNAi treatment of *Se-AQP* on nodulation and phenoloxidase (PO) activity. (**A**) Inhibitory effect of dsAQP on hemocyte nodule formation in response to bacterial challenge. One microliter of heat-killed (HK) *E*. *coli* (~4.2 × 10^4^ cells/larva) were injected into larvae at 24 h after dsRNA treatment. At 8 h PI, numbers of nodules were assessed. (**B**) Inhibitory effect of dsAQP on PO activity in response to bacterial challenge. One microliter of heat-killed (HK) *E*. *coli* (~4.2 × 10^4^ cells/larva) was injected into larvae at 24 h after dsRNA treatment. At 8 h PI of bacteria, PO activity was measured. A GFP gene was used as a control dsRNA (‘dsCON’). Each treatment was independently replicated three times. Each replicate used 10 larvae. Different letters indicate significant differences among means at Type I error = 0.05 (LSD test).

In response to a large number of bacteria, hemocytes induced around 62 nodules per larva in *S*. *exigua* (Fig. 7A). DEX treatment along with bacterial infection significantly (*P* < 0.05) inhibited nodule formation. Addition of PGE_2_ significantly (*P* < 0.05) rescued such immunosuppression. Decrease of *Se-AQP* expression by RNAi significantly (*P* < 0.05) impaired nodule formation in response to bacterial challenge. Addition of PGE_2_ failed to rescue such immunosuppression.

PO activity is required for nodule formation by catalyzing melanization to produce black nodules. It can be induced by the release of its precursor from oenocytoids through cell lysis after water influx^26,27^. Oenocytoid cell lysis resulted in PO activation via PG signaling as seen in PGE_2_ treatment (Fig. 7B). However, *Se-AQP* expression decreased by RNAi treatment prevented hemocytes from responding to PGE_2_ and inducing PO activation even under immune challenge.

### Adverse effect of Se-AQP RNAi on immature development

**Figure 8:**
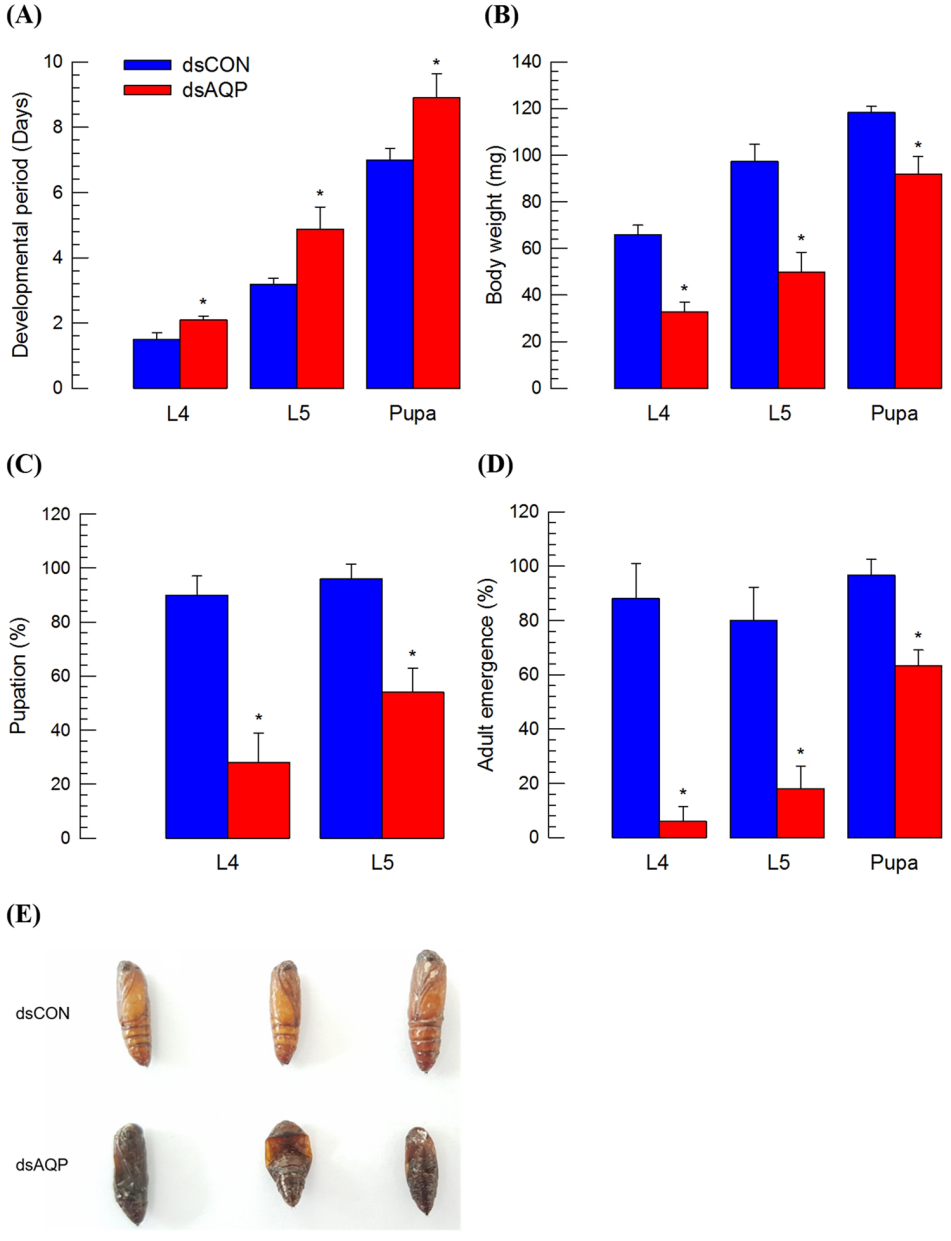
Influence of RNAi treatment of *Se-AQP* on larval and pupal developmental processes. One µg of dsCON or dsAQP was injected into larvae (within an hour after emerging into L4 and L5) or pupae (<4 h old) using a microsyringe. (**A**) Effect of dsAQP on developmental period of larvae and pupa. (**B**) Effect of dsAQP in decreasing body weights of larvae and pupa. (**C**) Pupation percentage in larvae after treatment with dsRNA. (**D**) Percentage of successful adult emergence in larvae and pupa after RNAi. (**E**) Detrimental effect of dsAQP on pupa. A GFP gene was used as a control dsRNA (‘dsCON’). Each treatment was independently replicated three times. Each replicate used 10 larvae. Asterisk mark (*) on bars indicates significant differences among means at Type I error = 0.05 (LSD test).

Transport of water or small molecules through Se-AQP might be necessary for development of *S*. *exigua*. To test this hypothesis, larval and pupal developments of *S*. *exigua* were monitored to compare any difference between RNAi-treated and control individuals (Fig. 8). For larval instars L4 and L5 and pupae, developmental rates were significantly (*P* < 0.05) retarded after RNAi treatment to decrease *Se-AQP* expression. RNAi-treated individuals exhibited 1.11∼1.40 folds slower developmental rates compared to control (Fig. 8A). RNAi treatment also significantly reduced body size, resulting in only half size of larvae or 86% body weight of pupae compared to control (Fig. 8B). Adverse effects of RNAi treatment resulted in significant (*P* < 0.05) mortalities at larval (Fig. 8C) and pupal (Fig. 8D) stages. In addition, RNAi treatment resulted in malformed pupae. They could not emerge to adults (Fig. 8E).

## Discussion

An aquaporin gene, *Se-AQP*, of *S*. *exigua* was identified in this study. Its molecular structure has characters of other insect-specific AQPs. All developmental stages of *S*. *exigua* expressed *Se-AQP*. However, its expression varied with environmental conditions. Especially, its physiological function of transporting water and other small solutes allowed cell shape change of hemocytes to perform cellular immune responses. In addition, its expression was required for development of immature stages. This conclusion is supported by the following observations.

First, Se-AQP was homologous to insect DRIP type AQPs. Insect AQPs are composed of four distinct groups^33^. Group 1 AQPs include DRIP and PRIP subgroups, both of which are widespread in insects^34,35^. They have been characterized primarily in liquid-feeding insects such as green leafhopper^36^, buffalo fly^37^, yellow fever mosquito^38^, pea aphid^39^, whitefly^40^, and pest bugs^41^. In each of these species, DRIP is distributed mostly in hindgut (HG) and Malpighian tubule (MT). Mosquitoes also express PRIP in digestive and excretory tissues^42,43^. Both types of AQPs are water-specific^44^. They are presumed to perform osmoregulation of sap-feeding insects by extruding excess amounts of water or avoid salt overload through dietary intake^45^. Groups 2 and 4 AQPs are known as BIB and superaquaporins, respectively^6,46^. Group 3 AQPs are heterogeneous. Some of them have been characterized as Glp or Eglp that can transport glycerol in addition to water^8,47–49^. Transportation of water molecule through AQP is accomplished by projecting opposing NPA motifs containing two inverted helices on loop B and loop E^50^. Some insect AQPs can achieve glycerol transportability when they lose the first NPA classical motif ^51^. Transmembrane domain and structural analysis showed that Se-AQP had conserved characteristics of water-transporting activity. In vertebrate AQPs, Ar/R constriction region is composed of four conserved residues: Phe-58, His-182, Cys-191, and Arg-197^52^. In Se-AQP, this motif is conserved except that Cys-191 is replaced by Ser-226. Similar phenomenon has been observed for an AQP of *Chilo suppressalis* where Cys-191 is replaced by Ser-203^53^. Comparative analysis between vertebrate and insect AQPs has indicated that vertebrate Cys-191 is substituted by either Ser or Ala in insect DRIPs^53,54^. These bioinformatics analyses suggest that Se-AQP is insect-specific. It may perform water transportation.

Second, *Se-AQP* was expressed in all developmental stages from egg to adult. In larval stage, Se-AQP was expressed in all tested tissues including hemocytes. In other insects, different AQPs exhibit variations in their expression levels depending on development stages. For example, in *Culex pipiens*, mRNA levels of DRIP, Eglp, and AQP12L are significantly higher in larvae compared to those in pupae and adults whereas BIP mRNA levels are significantly lower in larvae compared to those in pupae and adults^55^. In contrast, in another mosquito species (*Anopheles gambiae*), mRNA levels of both PRIP and Eglp1 are similar between larvae and pupae^51^. Relatively low expression of AQP in fat body might be due to low AQP frequency in this tissue compared to its abundance in other tissues as seen for known AQPs in other insects. DRIPs are known to be present in fat body at low abundance^17,33^. In contrast, gut exhibited high expression of *Se-AQP*. This may be explained by crucial roles of gut in water homeostasis and osmoregulation carried out by two its components: HG and MT^56^. Likewise, the high expression level of *Se-AQP* in hemocytes suggests a high transporting activity of water and other small molecules through Se-AQP of hemocytes.

Variation in *Se-AQP* expression levels was observed in *S*. *exigua* having different body weights with the same developmental and environmental factors (ambient temperature and humidity). High correlation between body weight and expression levels of *Se-AQP* suggests that AQP is associated with metabolic activity. In *Bombyx mori*, DRIP and PRIP AQPs are expressed during egg development^57^. Although no specific locality is observed for PRIP AQPs, DRIP AQPs are localized in peripheral yolk granules in diapause-destined eggs during transition from vitellogenesis to choriogenesis while they are evenly distributed among medulla yolk granules in nondiapause-destined eggs. Furthermore, DRIP AQPs in diapause-destined eggs are inert, supporting the association of AQPs with metabolism. Increase of Se-AQP expression with ambient temperature may be explained by the increase in metabolic rate. The plasticity of expression level of *Se-AQP* may help *S*. *exigua* adapt to extreme conditions of relative humidity. In *A*. *gambiae*, dry conditions with relative humidity less than 20% can lead to a significant reduction in AQP gene expression to prevent water loss from the body^58^.

Third, RNAi treatment against Se-AQP impaired maintenance of hemocyte cell shape under different osmotic pressure and prevented up-regulation of hemolymph glycerol titer under RCH. The failure of hemocyte shape change under different osmotic pressure suggests inefficient water flux of hemocyte membrane due to knocking down of *Se-AQP* expression. This suggests that Se-AQP plays a role in water transportation of *S*. *exigua*. RCH did not up-regulate hemolymph glycerol titer in RNAi-treated larvae against Se-AQP, suggesting that Se-AQP might also transport glycerol. This is supported by the prediction of protein interaction between Se-AQP and glycerol kinase. *S*. *exigua* is classified as freeze-susceptible insect that possesses supercooling capacity^59^. Supercooling capacity can be achieved by accumulating high amounts of polyols or other forms of cryoprotectants^60^. In *S*. *exigua*, glycerol is accumulated during RCH, in which glycerol kinase catalyzes conversion of dihydroxyacetone-3-phosphate into glycerol^32^. Thus, under RCH, glycerol is produced by glycerol kinase near to Se-AQP, through which the newly synthesized glycerol may be secreted and accumulated in the hemolymph. However, RNAi treatment against *Se-AQP* expression prevented the glycerol movement into hemolymph. Thus, the glycerol titer in hemolymph was not up-regulated by RNAi treatment. This suggests that Se-AQP may play a crucial role in redistribution of water content in insects during RCH to tolerate freezing temperatures. In freeze-tolerant insect *Eurosta solidaginis* (goldenrod gall fly), AQPs appear to coordinate redistribution of water and glycerol transportation because mercuric chloride (a specific inhibitor against AQP activity) can prevent the freeze tolerance of this fly^61^.

Fourth, AQP modulated cell shape change of hemocytes by mediating their spreading behavior because hemocytes after AQP RNAi treatment failed to exhibit spreading behavior. Hemocyte-spreading behavior that extends cytoplasm in specific directions is required for cellular immune responses such as phagocytosis and nodule formation. It is triggered by several immune mediators for actin rearrangement^62,63^. It has been suggested that transmembrane water fluxes through AQPs play pivotal roles in cell shape change via local dilution by water along with actin polymerization^64^. In neural astrocytes, AQP4 activity is required for cytoplasmic extension along with F-actin growth^65^. Thus, the water transporting activity of Se-AQP is likely to be associated with hemocyte-spreading behavior. The role of Se-AQP in cell shape change of hemocytes was further supported by immunosuppression induced by RNAi treatment, resulting in significant impairments in phagocytosis and nodulation upon bacterial challenge or PGE_2_ treatment. Furthermore, PO activity was significantly reduced by RNAi treatment against Se-AQP. This is because its inactive precursor (PPO) is produced from oenocytoids and released to plasma via cell lysis to be activated by proteases^26,66^. A specific receptor for PGE_2_ can mediate cell lysis by activating sodium-potassium-chloride cotransporter to facilitate water influx^27^. These observations support the physiological role of Se-AQP in cellular immune responses by changing cell shape through transmembrane water fluxes.

Finally, RNAi treatment against *Se-AQP* expression resulted in developmental retardation and alteration of *S*. *exigua* immatures. Early intervention by RNAi treatment was much detrimental to later development to adults as seen in L4 larval treatment, resulting in the least percentage rate of adult emergence. This suggests that RNAi treatment can increase the severity, resulting in detrimental effect. Similar detriment effects of RNAi against AQP genes have been reported in *Tribolium castaneum* that expresses nine AQPs^67^. Knockdown of *TcEglp3*, *TcEglp4*, or *TcDRIP* killed ~20% to ~60% of larvae before pupation^67^. Because most AQPs of *T*. *castaneum* are expressed in HG and MT, the lethal effect of RNAi treatment can be explained by malfunctioning of excretory system^67^. This suggests that the high mortality observed in *S*. *exigua* treated by RNAi against *Se-AQP* expression might be also due to malfunctioning of excretory system in HG and MT where *Se-AQP* is likely to be highly expressed. This supports the prediction that AQPs might act as a potential molecular target for insect pest management^39,44,68^.

In summary, this study reports the first AQP of *S*. *exigua* with its gene structure and physiological functions. Its transporting function of water and small molecule is crucial for cell shape change which is required for cellular immune responses of hemocytes and development. The fact that hemocyte cell shape change induced by PGE_2_ treatment is prevented by RNAi of *Se-AQP* expression suggests that PG signaling is functionally associated with Se-AQP activity and cytoskeletal rearrangement. This opens a new area of PG signal transduction pathway that needs to be explored in a subsequent study.

## Methods

### Insect rearing and bacterial culture

Rearing of *S*. *exigua* followed published method^31^. Under rearing conditions, larval stage lasted about 13 days from first instar (L1) to fifth instar (L5) before pupation. *Escherichia coli* Top10 (Invitrogen, Carlsbad, CA, USA) was cultured in Luria-Bertani (LB) medium (BD, Franklin Lakes, NJ, USA) overnight at 37 °C with shaking at 180 rpm. For immune challenge, bacteria were heat-killed at 95 °C for 10 min. The number of bacterial cells was then counted using a hemocytometer under a phase contrast microscope (BX41, Olympus, Tokyo, Japan).

### Chemicals

Prostaglandin E_2_ (PGE_2_: (5Z,11α,13E,15 S)-11,15-dihydroxy-9-oxoprosta-5,13-dienoic acid), dexamethasone (DEX: (11ß,16α)-9-fluoro-11,17,21-trihydroxy-16-methylpregna-1,4-diene-3), and L-dihydroxyphenylalanine (DOPA) were purchased from Sigma-Aldrich Korea (Seoul, Korea) and dissolved in dimethyl sulfoxide (DMSO). Fluorescein isothiocyanate (FITC)-tagged phalloidin (Alexa Fluor 488 phalloidin) and 4′,6-diamidino-2-phenylindole (DAPI) were purchased from Thermo Fisher Scientific Korea (Seoul, Korea). Anticoagulant buffer (ACB) was prepared in 186 mM NaCl, 17 mM Na_2_EDTA, and 41 mM citric acid. Its pH was then adjusted to 4.5 with HCl.

### Bioinformatics and sequence analysis

*Se-AQP* sequence was analyzed using Lasergene EditSeq program (Ver. 7.1, DNASTAR, Madison, WI, USA) to predict open reading frame (ORF) and amino acid sequence. Its ORF sequence was deposited at GenBank with accession number of MH333284. Phylogenetic analysis was performed using MEGA6. Transmembrane domains of Se-AQP were predicted using TMHMM^69,70^. UCSF Chimera (https://www.cgl.ucsf.edu/chimera/) was used for protein motif analysis. Protein-protein interaction map was generated using STRING 10.0a (http://version10a.string-db.org).

### Heterologous expression of *Se-AQP* in Sf9 cells

Using an eukaryotic expression vector pIB/V5-His (Invitrogen), a recombinant pIB/V5-His-*Se-AQP* was prepared and transiently expressed in Sf9 cell line by cationic lipid-mediated transfection using X-treme GENE 9 DNA transfection reagent (Roche, Mannheim, Germany). Transfection procedure and extraction of cellular proteins from Sf9 cells were performed according to the method described by Kumar and Kim^23^ and quantified using Bradford method^71^.

### Western blotting

Extracted proteins (~100 µg/sample) were separated on 10% SDS-PAGE and subjected to western blotting according to the method described by Kumar and Kim^23^.

### RNA extraction and RT-PCR

Total RNAs were extracted from different developmental stages of *S*. *exigua* using approximately 500 eggs, 30 individuals for L1 or L2, 5 individuals for L3 or L4, and one individual for L5 as an experimental unit (EU). To extract total RNAs from different tissues of L5 larvae, 3-day old L5 (L5D3) larvae were dissected in PBS. By cutting a proleg, hemolymph was collected while the remaining body was used to isolate fat body and gut. The collected hemolymph in ACB was centrifuged at 800 × *g* for 5 min. The resulting hemocyte pellet was used to extract total RNA with Trizol reagent (Invitrogen) according to the manufacturer’s instruction. After DNase treatment, RT-PCR was performed following the method described by Kumar and Kim^23^ with gene-specific primers (Table S1). Quantitative PCR (qPCR) was performed according to the general guideline suggested by Bustin *et al*.^72^. Ribosomal protein RL32 gene was used as a stably-expressed reference gene for qPCR with gene-specific primers (Table S1)^73^. Each treatment was replicated three times using independent RNA collections. Quantitative analysis of gene expression was done using the comparative CT (2^−ΔΔCT^) method^74^.

### Humidity and temperature stress treatment

Three developmental stages (L4, L5, and pupa) after environmental stress treatment were analyzed and individual was assigned to each EU. For temperature stress assessment, each test individual was confined in a glass tube (25 × 50 mm) and exposed to different temperatures (10°, 16°, 20°, 25°, and 37 °C) for 6 h under 60 ± 10% RH. For humidity treatment, each test individual was kept in a small vented insect rearing box (73 × 73 × 73 mm) with different RH (10, 25, 60, 75, and 90%) in desiccators (ThermoFisher Scientific Korea) placed at 25 °C for 24 h. Different RH levels were prepared following Rockland method^75^. Each treatment was replicated three times.

### Glycerol quantification in hemolymph and rapid cold hardening (RCH) bioassay

Hemolymph (~150 µL) from L5 larvae was collected into 1.7 mL tube containing 350 µL of ACB. Subsequent HPLC analysis followed the method described by Park and Kim^32^. For RCH bioassay, L5D3 larvae were randomly selected from the rearing stock. Test individuals were divided into two RNAi treatment groups. One group was exposed to 4 °C for 6 h prior to cold treatment (−10 °C for 1 h) while the other group was directly exposed to cold treatment without prior exposure to cool temperature. Subsequent bioassay followed the method described by Park and Kim^32^. Each treatment was replicated three times. Each replication used 10 individuals.

### RNA interference (RNAi) of Se-AQP expression

Template DNA was amplified with gene-specific primers (Table S1) containing T7 promoter sequence at the 5′ end. Double-stranded RNA (dsRNA) encoding *Se-AQP* (‘dsAQP’) or control dsRNA (‘dsCON’) was then prepared following method described by Vatanparast *et al*.^76^. After mixing with a transfection reagent Metafectene PRO (Biontex, Plannegg, Germany) in 1:1 (v/v) ratio, the mixture was then incubated at 25 °C for 30 min to form liposomes to increase RNAi efficiency. To prepare dsCON, 500 bp fragment of green fluorescent protein (GFP) gene was synthesized. In every experiment, 1 µg of dsAQP was injected into larva or pupa using a microsyringe (Hamilton, Reno, NV, USA) equipped with a 26-gauge needle. RNAi efficiency was determined by RT-qPCR against *Se-AQP* expression at 24 and 48 h post-injection (PI). Each treatment was replicated three times using independent RNA preparations.

### Immunofluorescence assay (IFA) for hemocyte-spreading behavior

IFA followed the method described by Kumar and Kim^23^. At 24 h PI of dsCON or dsAQP, L5 larvae was immune-challenged with 1 µL of heat-killed *E*. *coli* (~3.2 × 10^4^ cells/larva). One microliter of DEX (1 µg/µL) or PGE_2_ (1 µg/µL) was injected separately along with heat-killed bacteria. In all cases, at 2 h PI of bacteria, hemocyte-spreading behavior was checked under a fluorescence microscope at 400× magnification. Hemocyte-spreading was determined by the extension of F-actin out of the original cell boundary. Hemocyte-spreading behavior was quantified by randomly assessing 100 cells. Each treatment was replicated three times.

### Osmotic shock

At 24 h PI of dsCON or dsAQP, hemolymph was collected and fixed onto glass coverslip. After washing three times with PBS, cells were incubated with 10 µL of each of three different solutions including isotonic (TC100), hypertonic (10% glucose in TC100), and hypotonic (10 times diluted TC100 with deionized water) solutions for 10 min. After washing three times with PBS, cells were permeabilized with 0.2% Triton X-100 in PBS for 2 min at RT and subjected to IFA as described above. Hemocyte-spreading behavior was quantified as described above. Shrunk cells and hemocyte lysis were quantified separately by randomly checking 100 cells. Each treatment was replicated three times with independently prepared biological samples.

### Phagocytosis

FITC-labeled *E*. *coli* were prepared with a general antibody-labeling method using ammonium chloride^77^. After confirmation of tagging under microscope, bacteria (1 µL) (~3.5 × 10^4^ cells/larva) were injected to each L5D3 larva at 24 h PI of dsCON or dsAQP. After 10 min, hemolymph from each EU (5 larvae) was collected. Hemocytes were then collected in ACB as mentioned earlier and centrifuged at 180 × *g* for 2 min at 4 °C. These hemocytes were then washed three times in ice-cold PBS with 0.02% EDTA to stop phagocytosis and remove extracellular bacteria. The final cell pellet was resuspended in TC100 medium. IFA was then performed as described mentioned earlier except that F-actin of hemocytes was stained with 5% of Alexa Fluor 555 phalloidin (Invitrogen). The proportion of phagocytic cells was determined under a fluorescence microscope at 400× magnification.

### Nodulation assay

Nodule counts followed the method described by Vatanparast *et al*.^76^. At 24 h PI of dsCON or dsAQP, L5D3 larvae were immune-challenged with 1 µL of heat-killed *E*. *coli* (~4.2 × 10^4^ cells/larva). One microliter of DEX (1 µg/µL) or PGE_2_ (1 µg/µL) was injected separately along with bacteria. For all cases, at 8 h PI of bacteria, nodule formation was assessed. Each treatment used 10 larvae. Each treatment was independently replicated three times.

### Phenoloxidase (PO) enzyme assay

Plasma PO activity was determined using DOPA as substrate. Activity was measured following the method described by Shrestha and Kim^26^. At 24 h PI of dsCON or dsAQP, each L5D3 larva was immune-challenged with 1 µL of heat-killed *E*. *coli* (~4.2 × 10^4^ cells/larva). One microliter of DEX (1 µg/µL) or PGE_2_ (1 µg/µL) was injected separately along with heat-killed bacteria. For all cases, at 8 h PI of bacteria, PO activity was assessed. Each treatment consisted of three biologically independent replicates. Each replicate used 10 larvae.

### Developmental assay

Developmental period was defined as elapsed time in days from injection (one day old L4 larvae) to pupation. One µg of dsCON or dsAQP was injected into larvae (within an hour after emerging into L4 and L5) or pupae (<4 h old) using a microsyringe. Each treatment was replicated three times. Each replicate used ten insects.

## Supplementary information

Supplimentary data
