# Supplementary material for: An aquaporin mediates cell shape change required for cellular immunity in the beet armyworm, Spodoptera exigua
Source: Sci Rep. 2019 Mar 21;9:4988. doi: 10.1038/s41598-019-41541-2 (PMC6428837; doi:10.1038/s41598-019-41541-2)
Supplement: Supplementary file 1 — Supplimentary data [file 41598_2019_41541_MOESM1_ESM.pdf]

***Scientific Reports***

# An aquaporin mediates cell shape change required for cellular immunity in the beet armyworm, *Spodoptera exigua*

**Shabbir Ahmed & Yonggyun Kim**

Department of Plant Medicals, Andong National University, Andong 36729, Korea

Corresponding author: hosanna@anu.ac.kr

**Table S1.** Primers used in this study

| Primer | Orientation | Sequence (5´- 3´) | Purpose |
|---|---|---|---|
| *Se-AQP* | Forward | TGGCGATACCCGAAAACCAA | RT & qPCR |
| | Reverse | CTGGAGCCGGTGAATGGAAT | |
| | T7 Forward | TAATACGACTCACTATAGGGAGATG GCGATACCCGAAAACCAA | RNAi |
| | T7 Reverse | TAATACGACTCACTATAGGGAGACT GGAGCCGGTGAATGGAAT | |
| | ORF Forward | GAAATGGCAAGCAAGACTGTGAAC | Cloning into |
| | ORF Reverse | GAAGTCGTAGGAGCCGCTGTC | pIB/V5-His vector |
| *RL32* | Forward | ATGCCCAACATTGGTTACGG | Internal control for |
| | Reverse | TTCGTTCTCCTGGCTGCGGA | RT & qPCR |
| *GFP* | Forward | CGAGATGGTGAGCAAGGGCG | RNAi Control |
| | Reverse | GTACCTTACTTGTACAGCTC | |
| | T7 Forward | TAATACGACTCACTATAGGGAGACG AGATGGTGAGCAAGGGCG | |
| | T7 Reverse | TAATACGACTCACTATAGGGAGAGT ACCTTACTTGTACAGCTC | |

**Figure S1.** Three-dimensional structure of Se-AQP. Monomers were grouped as tetramers in the biological membranes as top view was shown here which was prepared using UCSF Chimera (https://www.cgl.ucsf.edu/chimera/). White balls and sticks inside each monomer represented the NPA domain. (D) The protein-protein interaction maps of Se-AQP with other proteins of *D. melanogaster*. The map was generated by the STRING 10.0a (http://version10a.string-db.org). Connections between spheres representing individual proteins were based on experimental data, co-expression and gene neighborhood.

**Figure S2.** Biotic or abiotic factors to modulate expression of *Se-AQP*. (A) Correlation of *Se-AQP* expression with body weights in fourth (L4), fifth (L5) instar larvae, and pupae. (B) Relative expression of *Se-AQP* under temperature stress. Larvae and pupa were exposed for 10$^{o}$C, 16$^{o}$C, 20$^{o}$C, 25$^{o}$C or 37$^{o}$C for 6 h. (C) Relative expression of *Se-AQP* under humidity treatments. Larvae and pupa were treated by 10, 25, 60, 75 and 90% humidity for 24 h. Each treatment was independently replicated three times.

**Figure S3.** RNA interference (RNAi) of *Se-AQP* in the fifth instar larvae. One µg of gene-specific dsRNA (‘dsAQP’) was injected into L5D1. A GFP gene was used as a control dsRNA (‘dsCON’). (A) Effect of RNAi on *Se-AQP* expression in indicated tissues of L5 larvae: hemocyte (‘HC’), fat body (‘FB’) and gut (‘Gut’). (B) Relative expression of *Se-AQP* in hemocyte, fat body and gut after 24 and 48 h PI. Each treatment was independently replicated three times. Asterisk mark (*) on the bars indicates significant differences among means at Type I error = 0.05 (LSD test).

(A)

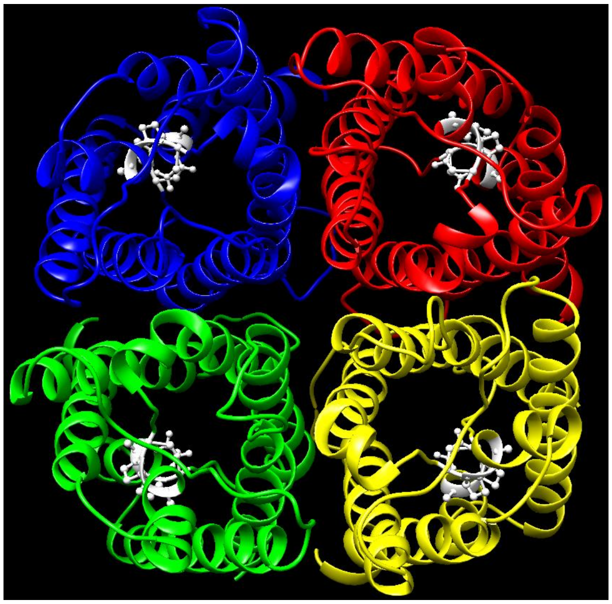

(B)

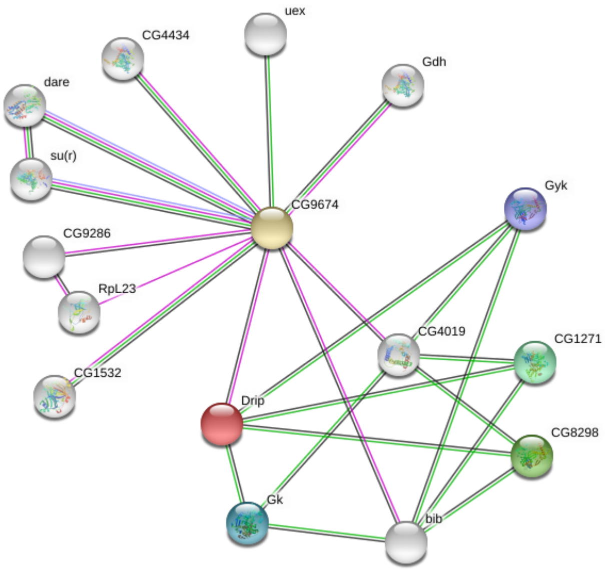
uex
CG4434
Gdh
dare
su(r)
CG9674
Gyk
CG9286
RpL23
CG4019
CG1271
CG1532
Drip
CG8298
Gk
bib

**Fig. S1**

(A)

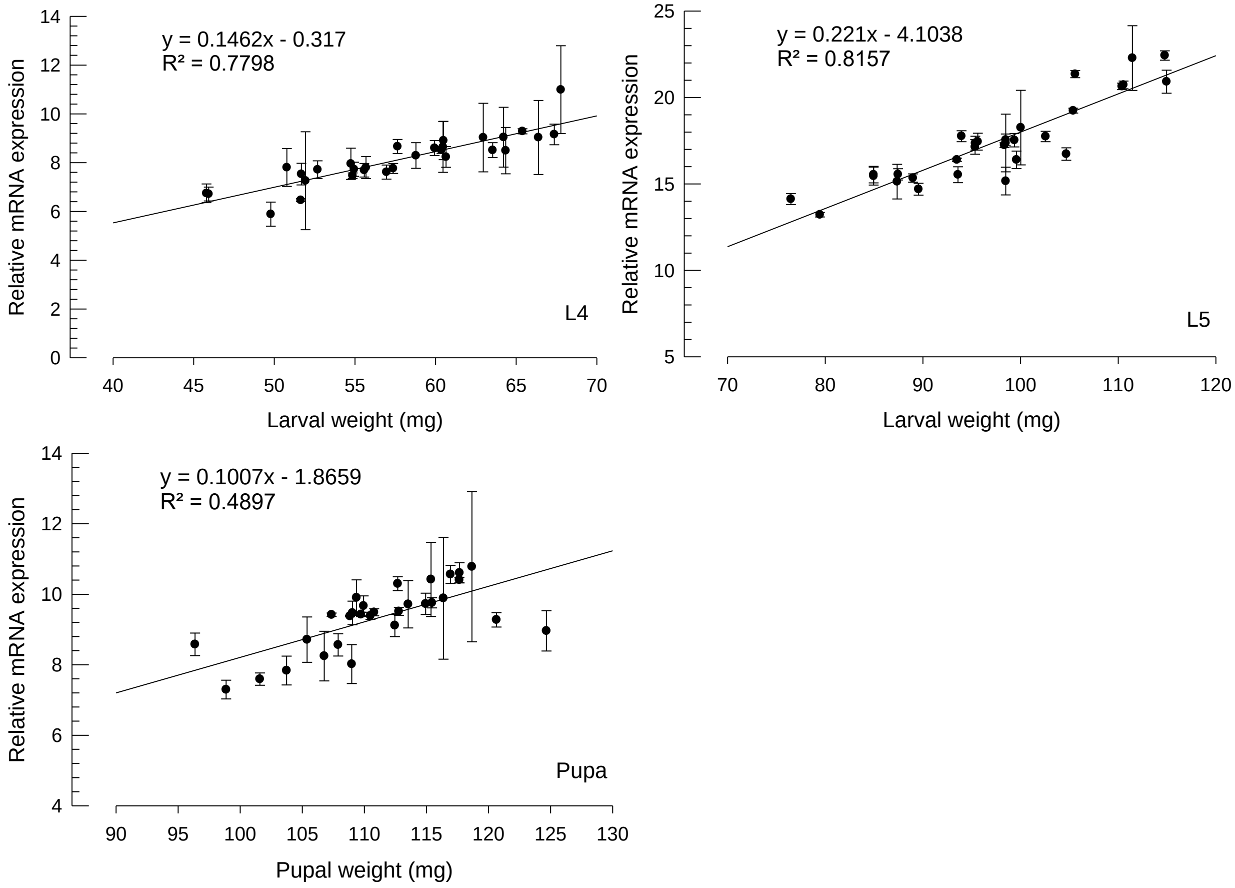
y = 0.1462x - 0.317
R² = 0.7798
L4
Relative mRNA expression
Larval weight (mg)
y = 0.221x - 4.1038
R² = 0.8157
L5
Relative mRNA expression
Larval weight (mg)
y = 0.1007x - 1.8659
R² = 0.4897
Pupa
Relative mRNA expression
Pupal weight (mg)

(B)

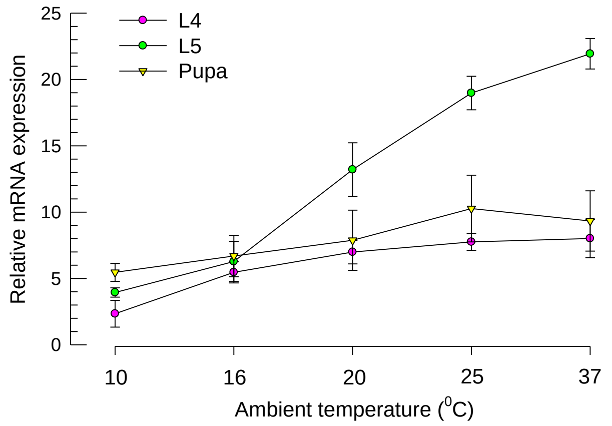
L4
L5
Pupa
Relative mRNA expression
Ambient temperature (0C)

(C)

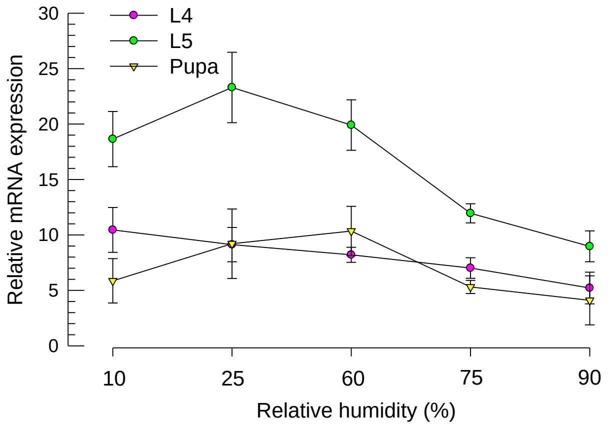
L4
L5
Pupa
Relative mRNA expression
Relative humidity (%)

**Fig. S2**

**(A)**

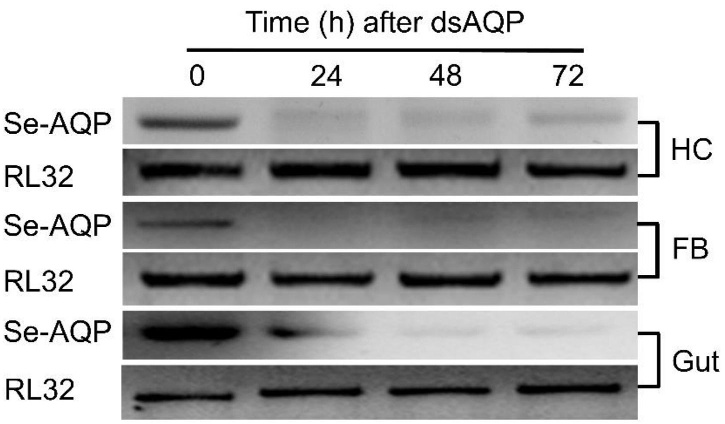

Time (h) after dsAQP
0
24
48
72
Se-AQP
RL32
HC
Se-AQP
RL32
FB
Se-AQP
RL32
Gut


**(B)**

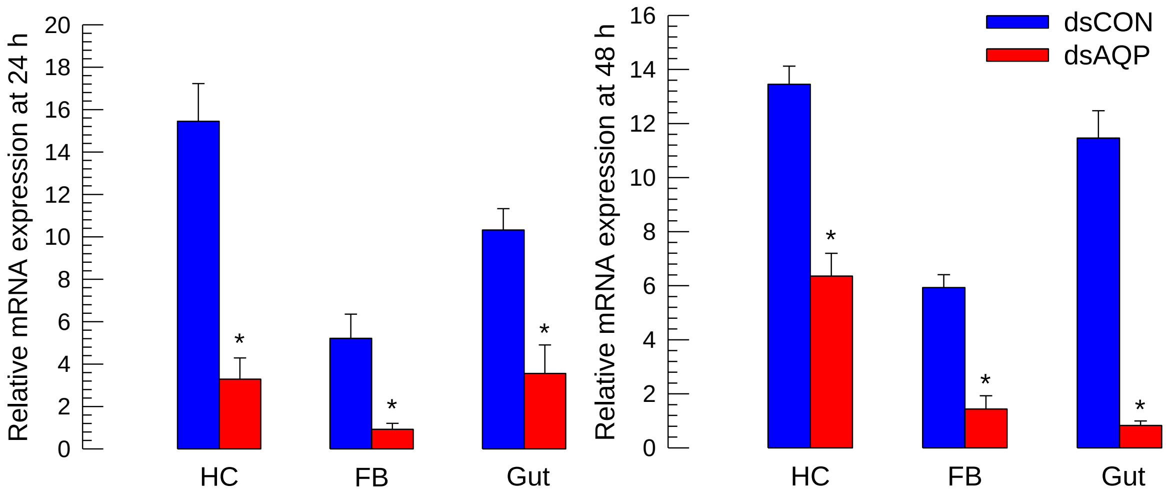

Relative mRNA expression at 24 h
Relative mRNA expression at 48 h
dsCON
dsAQP
HC
FB
Gut


**Fig. S3**
